# User Acceptance of Smart Home Emergency Response Systems: Mixed Methods Study

**DOI:** 10.2196/93003

**Published:** 2026-04-20

**Authors:** Michael Pantförder, Oliver Krüger, Annika Rietz, Colin Dzieia, Sven Meister

**Affiliations:** 1 Health Informatics, Faculty of Health, Witten/Herdecke University, Alfred-Herrhausen-Straße 50, Witten, 58455, Germany, 49 231 97677-437; 2 Healthcare Department, Fraunhofer Institute for Software and Systems Engineering, Dortmund, Germany; 3 Office of Fire, Rescue and Civil Protection, Dortmund, Germany

**Keywords:** smart home emergency response systems, SHERS, user acceptance, emergency response, mixed methods research, Double Diamond, structural equation modeling

## Abstract

**Background:**

Smart home emergency response systems (SHERS) leverage existing smart home infrastructure to detect critical events and alert emergency services without manual activation. Unlike personal emergency response systems, which require users to trigger alarms, SHERS initiate alerts autonomously. Although technically feasible, user acceptance has received limited empirical attention.

**Objective:**

This study examined factors influencing the intention to adopt SHERS in private households, identifying key facilitators and barriers to acceptance.

**Methods:**

A mixed methods study followed the Double Diamond framework. In the discover/define phases, expert interviews (n=3) and secondary data analysis informed persona and scenario development. In the “develop” phase, brainwriting workshops (6-3-5 method, n=12) generated design requirements translated into a low-fidelity prototype. In the “deliver” phase, an online survey (n=85) assessed acceptance using the Technology Usage Inventory. Structural equation modeling tested hypothesized relationships, and methodological triangulation integrated qualitative and quantitative findings.

**Results:**

Perceived accessibility was the strongest positive predictor of intention to use (β=0.33, *P*=.02), while skepticism showed a marginally negative effect (β=−0.34, *P*=.06). The model explained 66% of variance in behavioral intention (*R*²=0.66). Triangulation confirmed that concerns about complexity, false alarms, and data privacy underlie these effects. Experts emphasized that technology should support rather than replace human decision-making; workshop participants stressed intuitive setup and user control over alarm cancellation.

**Conclusions:**

SHERS acceptance is primarily associated with perceived accessibility, while skepticism may act as a barrier. Developers should prioritize seamless integration with existing ecosystems, clear feedback mechanisms to prevent false alarms, and strong data protection.

## Introduction

### Background

Emergency response systems usually assume that affected individuals can seek help by calling emergency services, activating alarm buttons, or evacuating dangerous areas [1]. However, physical limitations, cognitive impairments, or environmental factors often stop timely self-rescue. This is especially true for older adults living alone and people facing sudden medical issues [2]. In Germany, almost 6 million people aged 65 years and older live in single-person households [3], which puts a significant number at risk of delays in emergency response.

Smart home technologies provide a technical basis for addressing these gaps [4]. Recent developments in ambient assisted living and health monitoring show that these systems can spot anomalies, track daily activities, and detect potential emergencies through patterns of inactivity or changes in the environment [5,6]. Building on these abilities, smart home emergency response systems (SHERS) represent a new type of safety-critical application that can automatically identify emergency situations and send alerts to emergency services, caregivers, or nearby responders without requiring users to take action [7].

Such systems may draw on heterogeneous sensor configurations already present in smart home environments, including motion and presence sensors, environmental detectors for smoke or carbon monoxide, pressure-based bed and chair sensors that track prolonged inactivity, and wearable devices capable of detecting falls or abnormal vital signs [5,6]. By combining data from multiple sources, SHERS can distinguish routine patterns from potential emergencies and initiate alerts accordingly.

Unlike traditional personal emergency response systems that rely on manual activation, such as wearable alarm buttons [1], SHERS change the approach from user-initiated to system-initiated emergency management. When individuals cannot act, automated detection can close the delay between event and response and may help reduce mortality [8].

### Related Work

Research on smart home applications for health and safety has grown considerably, with ambient assisted living and telecare studies showing that sensor-based monitoring can support older adults [1,9-11]. Intelligent fire-detection systems have shown improved accuracy over standalone smoke detectors [12,13]. However, most current implementations operate as closed-loop systems that alert residents locally but still rely on human action to contact emergency services.

Behavioral research shows that people may fail to hear alarms, underestimate risks, or attempt to resolve emergencies independently [14-18]. These behavioral bottlenecks show that systems must be able to act autonomously when human response is delayed or absent.

At the infrastructure level, frameworks such as the Internet of Emergency Services [19] and smart emergency response systems [20] focus primarily on professional responders rather than end-user integration.

Technology acceptance research identifies usefulness, ease of use, trust, and privacy as key determinants of smart home adoption [21]. Similar patterns have been observed specifically for health-related smart home applications, where concerns about data security and loss of autonomy can inhibit adoption even when functional benefits are recognized [22]. However, safety-critical automation that acts on behalf of users in emergencies remains underexplored.

### Research Gap

Despite the technical feasibility of SHERS, empirical evidence on how users perceive *automated* emergency notification remains limited [23]. Three specific gaps emerge from the literature:

First, acceptance research rarely addresses safety-critical automation, where concerns about false alarms and privacy may differ from convenience-oriented applications [1,9,24].

Second, most studies examine acceptance factors in isolation rather than modeling their joint influence on behavioral intention. Understanding how psychological dispositions such as technology anxiety and curiosity, functional factors such as physical independence, and social factors such as prosocial orientation interact to shape adoption intentions requires multivariate approaches.

Third, the perspective of emergency professionals is rarely integrated into the design process.

### Objectives

This study addresses these gaps by investigating the adoption and intention to use SHERS in private households. Guided by a user-centered design approach, it pursues three objectives:

1. *Discover and define* the operational context, user needs, and design requirements for SHERS through expert interviews with emergency professionals and scenario-based design workshops.
2. *Develop and evaluate* a system concept that integrates qualitative insights into concrete design artifacts (personas, scenarios, and low-fidelity prototypes).
3. *Assess the determinants* of user acceptance through a quantitative survey examining how psychological, functional, and social factors influence intention to use.

By triangulating qualitative and quantitative findings within a structured design framework, this study seeks to provide both conceptual guidance for SHERS development and empirical evidence on the factors shaping public acceptance of automated emergency technologies.

## Methods

### Research Approach

This study adopted a user-centered design approach [25] operationalized through the Double Diamond framework [26], which structures innovation processes into 4 iterative phases: discover, define, develop, and deliver. This framework involves different user groups to ensure solutions reflect actual needs [27,28].

The study combined qualitative and quantitative methods across the 4 phases in a sequential exploratory design [29]. The reporting of this mixed methods approach is informed by the Mixed Methods Article Reporting Standards (JARS-MMARS) [30], which provide guidance on transparently documenting how qualitative and quantitative components are integrated. By synthesizing insights from expert interviews, participatory design workshops, and a structured acceptance survey, findings from each phase informed and validated subsequent stages.

**Figure 1.**
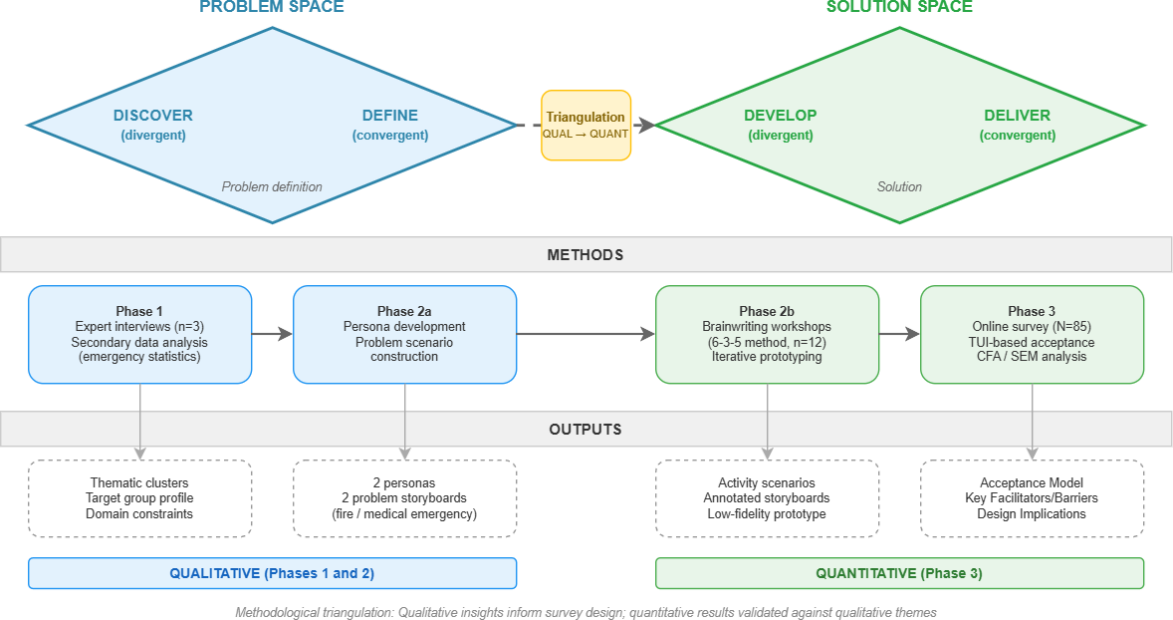
Overview of the Double Diamond research design with methods and outputs per phase. CFA: confirmatory factor analysis; SEM: structural equation modeling; TUI: Technology Usage Inventory.

Figure 1 illustrates the research design, mapping each phase to its corresponding methods, outputs, and how findings were iteratively integrated.

### Ethical Considerations

The ethics committee of Witten/Herdecke University approved the research project on September 7, 2022 (protocol code: S-152/2022). All participants provided informed consent prior to participation. For the online survey, consent was obtained electronically on the first page of the questionnaire. All data were collected anonymously; no personally identifiable information was stored. No financial or material incentives were offered for participation in any phase of this study.

### Phase 1: Discover—Domain Analysis and Problem Identification

The “discover” phase aimed to understand the operational context of emergency response and identify user groups who would benefit most from smart home-based emergency notification. This phase combined qualitative contextual inquiry with secondary data analysis.

#### Expert Interviews

We conducted 3 semistructured interviews with key stakeholders across the emergency response chain: a paramedic, a fire department unit leader, and a control center dispatcher. All 3 experts were affiliated with the Dortmund Fire Department, which served as the operational partner in the ADLeR (Automatisiertes Detektions-, Melde- und Leitsystem für Rettungskräfte [Automated Detection, Notification, and Guidance System for Emergency Responders]) project. Participants were purposively selected to represent the 3 core operational perspectives along the emergency response process: emergency dispatch and coordination, tactical incident management, and frontline medical care. This maximum-variation approach prioritized structural coverage over the number of interviews. As the interviews served primarily exploratory purposes within the “discover” phase, the resulting themes were subsequently validated and expanded through 2 participatory workshops with 12 additional participants (phase 2).

Following a problem-centered approach [31], interviews explored challenges in alerting, communication, and decision-making under uncertainty. Sessions were conducted using a virtual whiteboard, enabling participants to cocreate digital mind maps that captured workflows, pain points, and preliminary solution ideas. This participatory method aligns with principles of contextual design [32].

Interview data were thematically clustered by 2 researchers (OK and MP) to identify cross-cutting needs and constraints. Each researcher independently reviewed the mind map outputs and assigned thematic labels. Discrepancies were resolved through discussion until consensus was reached. As this step served primarily exploratory purposes, detailed COREQ (Consolidated Criteria for Reporting Qualitative Research) [33] reporting was not applied; however, the interview guide and mind map outputs are provided in Multimedia Appendix 1.

#### Secondary Data Analysis

To complement the qualitative findings, descriptive analysis was conducted using publicly available demographic data and anonymized emergency call statistics. The emergency data were provided by the Fire and Rescue Service of the City of Dortmund (Germany) and comprised all medically induced emergency calls recorded in 2021 (n=19,200). The dataset included age group classifications but contained no personally identifiable information. We analyzed age-stratified distributions to identify high-incidence population segments and inform target group selection for subsequent design activities.

#### Phase 1 Output

The combined findings provided a domain-grounded foundation for phase 2, including identified user needs, operational constraints, and a validated target group profile.

### Phase 2: Define and Develop—Scenario-Based Design and Prototyping

Building on insights from phase 1, phase 2 used scenario-based design techniques [34] to translate empirical findings into concrete design artifacts. This phase corresponds to both the “define” stage (problem framing through personas and scenarios) and the “develop” stage (solution ideation through participatory workshops) of the Double Diamond model.

#### Persona and Scenario Construction

Based on the thematic clusters from expert interviews and demographic indicators from the statistical analysis, we developed 2 representative user personas, capturing attributes such as age, living situation, health status, and technology familiarity. These personas guided the creation of 2 problem scenarios, a residential fire and a fall-related medical emergency, representing prototypical high-risk situations. Each scenario was visualized through low-fidelity storyboards depicting the current emergency response process without technological intervention, highlighting behavioral bottlenecks and communication gaps.

#### Participatory Design Workshops

We conducted 2 brainwriting workshops to validate and expand the initial scenarios. Brainwriting is a structured written ideation technique distinct from verbal brainstorming. Participants independently generate ideas in writing before passing them to others for elaboration, which reduces dominance effects and encourages balanced contributions [35]. We applied the 6-3-5 variant, in which 6 participants each write 3 ideas within 5 minutes, then rotate sheets for iterative refinement.

Workshop 1 involved 6 emergency professionals (first responders and dispatch personnel) who reviewed the problem scenarios and contributed design ideas using the 6-3-5 method. The session focused on identifying pain points, desired system behaviors, and integration requirements.

Workshop 2 involved 6 domain experts from academic and applied research institutions with expertise in aging societies, human-machine interaction, and sociotechnical systems. This session addressed system-level considerations, including usability, information interpretability, and edge-case behavior.

Based on workshop outputs, the problem scenarios were iteratively transformed into activity scenarios incorporating envisioned system functionalities. These were synthesized into a low-fidelity prototype combining key system elements into a coherent, user-facing concept.

#### Data Analysis

We conducted workshop sessions online using digital whiteboards. Exported materials served as the primary qualitative data source. Two researchers (AR and MP) independently analyzed the content following qualitative content analysis principles, inductively coding idea clusters to identify recurring patterns and design requirements. Discrepancies were resolved through iterative discussion until consensus was reached. Reporting follows the COREQ guideline [33]; details are provided in Multimedia Appendix 2.

#### Phase 2 Output

Validated activity scenarios, annotated storyboards, and a low-fidelity prototype serving as stimulus material for the acceptance evaluation.

### Phase 3: Deliver—Acceptance Evaluation

The “deliver” phase evaluated user acceptance of the proposed system concept through a quantitative online survey, completing the Double Diamond cycle by testing the developed solution with end users.

#### Survey Design and Constructs

Survey constructs were based on the Technology Usage Inventory (TUI) [36], which extends traditional acceptance models by incorporating individual traits including curiosity (NEU), anxiety (ANG), interest (INT), perceived usefulness (NÜT), user-friendliness (BEN), skepticism (SKE), and accessibility (ZUG). Abbreviations reflect the original German instrument. In the TUI framework, accessibility refers to the perceived availability of and ease of access to the technology, including how readily it can be obtained, set up, and integrated into daily routines. This construct does not refer to disability-related accessibility in the sense of barrier-free design. Two additional validated measures were included: the social compatibility (SOC) scale [37] for prosocial attitudes and the activities of daily living (ADL) scale [38] for functional independence.

The survey used a pre-post design: participants first completed demographic questions, smart home experience items, and baseline constructs (NEU, ANG, ADL, and SOC). They were then exposed to visual storyboards illustrating the emergency scenarios with integrated smart home functions (developed in phase 2). Post-exposure, participants rated the remaining TUI constructs and intention to use (ITU). This design enabled measurement of both general dispositions and concept-specific evaluations.

A pilot test (n=5) ensured item clarity and technical robustness. Survey implementation followed CHERRIES (Checklist for Reporting Results of Internet E-Surveys) reporting standards [39]; details are provided in Multimedia Appendix 3.

#### Participants

The survey was implemented as an open online questionnaire using LimeSurvey, hosted on a secure university server. It was distributed between October 2022 and June 2023 through multiple channels: housing cooperatives and senior advisory councils served as the primary recruitment partners, and the Dortmund Fire Department shared the survey link via social media (X, Facebook, and LinkedIn). Participants were also able to share the link independently. No financial or material incentives were offered. The only inclusion criterion was the ability to complete the German-language questionnaire; no further restrictions regarding age, health status, or technology experience were applied. A total of 128 individuals accessed the survey, of whom 85 completed all 4 sections (completion rate: 66%). Cookies were used to prevent repeated participation from the same device. The final sample included adults aged 20 to 89 years.

#### Statistical Analysis

We performed analyses in R (version 4.3.3) using lavaan (version 0.6‐20). We evaluated internal consistency using Cronbach α, with values ≥0.70 considered acceptable [40]. A 2-stage modeling strategy combined confirmatory factor analysis (CFA) and structural equation modeling (SEM). Given ordered-categorical measurement and the number of indicators per construct, a diagonally weighted least squares (DWLS) estimator with robust corrections was used. We evaluated model fit using scaled indices (chi-square, comparative fit index [CFI], Tucker-Lewis index [TLI], root mean square error of approximation [RMSEA], and standardized root mean square residual [SRMR]), with CFI/TLI >0.90 and RMSEA <0.08 interpreted as acceptable.

While no universally accepted minimum sample size exists for DWLS estimation, simulation research suggests that DWLS with robust corrections can yield adequate parameter estimates and fit indices at sample sizes below 200, particularly when indicators are ordinal and factor loadings are moderate to strong [41,42]. Nevertheless, with 85 participants and 9 latent predictors, the ratio of sample size to model complexity is limited, which may reduce statistical power for detecting moderate effects and increase the uncertainty of individual path estimates. The results should therefore be interpreted with appropriate caution regarding the precision of nonsignificant coefficients.

#### Phase 3 Output

Validated acceptance model identifying key facilitators and barriers to adoption intention.

### Methodological Integration and Triangulation

Findings from each phase directly informed subsequent stages. Specifically, the thematic clusters from expert interviews identified key concerns (such as false alarms, data privacy, and complexity of setup) that shaped the selection of acceptance constructs for the survey. The design requirements and activity scenarios generated in the workshops were translated into annotated storyboards that served as the stimulus material for the quantitative evaluation, ensuring that participants rated a concept grounded in domain-specific insights rather than an abstract technology description.

Methodological triangulation combined qualitative methods (interviews and workshops) with quantitative methods (survey and SEM), enabling cross-validation of findings. Table 1 summarizes the mapping of methods to phases and outputs.

**Table 1.** Mapping of Double Diamond phases to methods and outputs.

Phase	Double Diamond stage	Methods	Key outputs
1	Discover	Expert interviews (n=3) and secondary data analysis	Thematic clusters and target group identification
2a	Define	Persona development and problem scenario construction	2 personas and 2 problem storyboards
2b	Develop	Brainwriting workshops (12 participants) and iterative prototyping	Activity scenarios and low-fidelity prototype
3	Deliver	Online survey (n=85) and CFA^a^/SEM^b^ analysis	Acceptance model and design implications

a CFA: confirmatory factor analysis.

b SEM: structural equation modeling.

Following JARS-MMARS recommendations, the qualitative components (phases 1 and 2) adhere to COREQ reporting standards [33], while the quantitative component (phase 3) follows CHERRIES guidelines [39]. The integration of both strands occurred at multiple points: qualitative findings shaped the survey stimulus materials, and quantitative results were interpreted in the context of qualitative themes identified earlier. This sequential design meant that the acceptance evaluation was built on domain-specific insights instead of abstract constructs.

## Results

### Phase 1: Domain Analysis and Target Group Identification

#### Thematic Clustering From Expert Interviews

Across the 3 interviews conducted, a total of 82 individual idea nodes were cocreated using digital whiteboarding tools, resulting in 3 structured mind maps (Multimedia Appendix 1). Thematic clustering revealed recurring barriers and requirements along 3 operational layers: dispatch coordination, responder navigation, and lay responder integration.

A central theme concerned the limited situational awareness available to dispatchers at the time of call intake. Interviewees described frequent uncertainty due to missing contextual data, such as the caller’s precise location, environmental hazards, or patient-specific health risks.

First responders, in turn, reported considerable time losses caused by restricted access to buildings and unclear apartment layouts. Navigating unfamiliar environments, particularly under time pressure, was cited as a major operational burden. Participants noted that prior knowledge about access points, apartment configurations, or mobility limitations could significantly streamline response efforts.

Finally, the role of lay responders proved both promising and problematic. While early arrival can be beneficial, the lack of standardized guidance and limited integration into formal rescue coordination restricts their effectiveness. Suggestions included structured onboarding, risk-adapted task delegation, and access to context-specific information prior to arrival.

#### Demographic Analysis and Emergency Statistics

Descriptive analysis of age-stratified emergency statistics confirmed the relevance of older adults as a disproportionately affected population segment. Although individuals aged 60 years and above represent less than one-third of the general population, they accounted for 64% (12,232/19,200) of all medically induced emergency calls in 2021 (Table 2). This disproportionality indicates that age is not merely a proxy for population size but a significant factor in emergency system usage. Thus, older adults were selected as the primary target group for subsequent design phases.

**Table 2.** Age distribution of medical emergencies in domestic settings reported in Dortmund (Germany), a subset of total emergency calls in 2021 (N=19,200).

Age group (years)	Emergencies, n (%)
0‐9	666 (3.47)
10‐19	438 (2.28)
20‐29	1291 (6.72)
30‐39	1310 (6.82)
40‐49	1258 (6.55)
50‐59	2005 (10.44)
60‐69	2611 (13.60)
70‐79	3479 (18.12)
80‐89	4644 (24.19)
90‐99	1457 (7.59)
100‐110	41 (0.21)

These findings emphasize the need for improved information availability, environmental transparency, and support mechanisms for nonprofessional actors, which directly informed the scenario development in phase 2.

### Phase 2: Scenario Development and Requirements Engineering

#### Findings From Scenario-Based Brainwriting

The structured idea generation yielded a diverse set of system-relevant concepts, which were subsequently clustered into key design implications. Participants emphasized the importance of real-time contextual awareness enabled by ambient and body-worn sensor data, precise geolocation, and adaptive alarm routing tailored to user roles and incident types. Suggestions also included infrastructure-level support for building access and orientation, such as dynamic lighting, illuminated door signage, and smart locking mechanisms.

Several contributions addressed systemic integration challenges, highlighting the need for interoperability across heterogeneous devices and platforms. The envisioned system was expected to balance automation with user override options and to provide differentiated access to sensitive data, depending on responder qualification and proximity. Participants also raised concerns regarding privacy, false-positive prevention, and reliability under edge-case conditions.

#### Scenario Iteration and Consolidation

Based on these inputs, we revised the initial scenarios into detailed activity scenarios incorporating technical functionalities and user interactions, visualized through annotated low-fidelity storyboards.

A subsequent workshop evaluated these scenarios, focusing on usability, privacy, and error tolerance. Feedback was synthesized into a low-fidelity prototype illustrating key components and user touchpoints.

**Figure 2.**
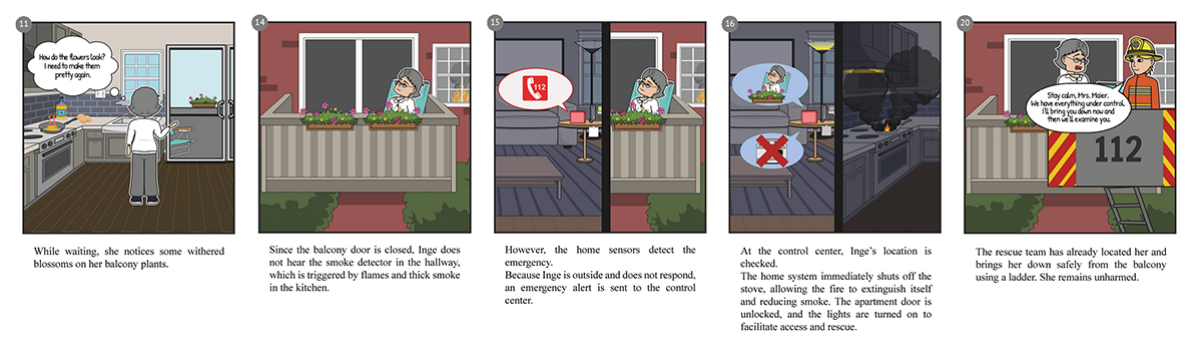
Selected panels from the fire emergency scenario illustrating autonomous system intervention.

Figure 2 illustrates selected panels from the fire emergency scenario, depicting the sequence from unnoticed hazard through autonomous detection, system-initiated intervention, and successful responder arrival (see Multimedia Appendix 2 for complete storyboards).

The resulting artifacts, including 29 design requirements and annotated storyboards (Multimedia Appendix 2), served as the conceptual foundation for the acceptance study conducted in phase 3.

### Phase 3: Evaluation and Statistical Analysis

#### Descriptive Analysis

A total of 85 participants completed all 4 sections of the survey. The sample included individuals aged 20 to 89 years, with the most represented groups being those aged 30‐39 years (n=23, 27%) and 20‐29 years (n=16, 19%). A majority of participants identified as male (n=55, 65%), and most lived either in one-person (n=28, 33%) or two-person households (n=36, 42%). Regarding education, 44% (n=37) reported a college degree, while 29% (n=25) held a secondary school qualification.

Current smart home usage was reported by 61% (52/85) of participants, with 33% (28/85) indicating an intention to adopt such technologies within the next 12 months. Adoption intentions varied slightly by age, gender, and living arrangement (see Table 3).

**Table 3.** Demographic distribution of participants and their smart home technology usage patterns, including current usage and future intentions.

	Count (n=85), n (%)	Current smart home usage, n (%)	Planned smart home adoption, n (%)
Total		52 (61)	28 (33)
Sex			
Female	30 (35)	15 (50)	5 (17)
Male	55 (65)	37 (67)	23 (42)
Age (years)			
20‐29	16 (19)	9 (56)	6 (38)
30‐39	23 (27)	14 (61)	9 (39)
40‐49	13 (15)	9 (69)	4 (31)
50‐59	12 (14)	8 (67)	5 (42)
60‐69	9 (11)	7 (78)	3 (33)
70‐79	8 (9)	4 (50)	1 (12)
80‐89	4 (5)	1 (25)	0 (0)
Education			
Intermediate school	25 (29)	12 (48)	4 (16)
High school	15 (18)	11 (73)	7 (47)
College	37 (44)	24 (65)	13 (35)
Doctorate/PhD	8 (9)	5 (62)	4 (50)
Living situation			
Single-person household	28 (33)	18 (64)	6 (21)
Two persons	36 (42)	23 (64)	13 (36)
Three or more persons	21 (25)	11 (52)	9 (43)

Descriptive statistics for all psychometric constructs and individual items are summarized in Table 4. Participants reported high levels of functional independence, with mean scores above 4.6 across all ADL items (eg, ADL1: mean 4.81, SD 0.72). Social compatibility showed moderate to high values (SOC1-SOC5: mean>4.2), though lower agreement was observed for items reflecting burdensome social responsibility (SOC6-SOC9: mean≈3‐4).

**Table 4.** Descriptive statistics of item responses.

Item	Mean (SD)
ADL (activities of daily living)	
ADL1	4.81 (0.72)
ADL2	4.68 (0.88)
ADL3	4.86 (0.54)
ADL4	4.66 (0.92)
SOC (social compatibility)	
SOC1	5.00 (1.02)
SOC2	4.49 (1.14)
SOC3	5.12 (1.07)
SOC4	4.95 (1.06)
SOC5	4.24 (1.26)
SOC6	3.01 (1.56)
SOC7	3.91 (1.57)
SOC8	4.18 (1.37)
SOC9	4.34 (1.34)
SOC10	4.34 (1.32)
NEU (curiosity)	
NEU1	6.04 (1.19)
NEU2	4.26 (1.96)
NEU3	5.72 (1.1)
NEU4	4.82 (1.72)
ANG (anxiety)	
ANG1	2.38 (1.58)
ANG2	2.31 (1.47)
ANG3	2.07 (1.32)
ANG4	2.01 (1.34)
INT (interest)	
INT1	5.58 (1.49)
INT2	4.86 (1.54)
INT3	4.88 (1.58)
INT4	4.96 (1.58)
NÜT (usefulness)	
NÜT1	5.99 (1.11)
NÜT2	4.31 (1.63)
NÜT3	4.84 (1.57)
NÜT4	4.31 (1.59)
SKE (skepticism)	
SKE1	4.20 (1.7)
SKE2	2.54 (1.45)
SKE3	2.34 (1.31)
SKE4	2.36 (1.28)
BEN (user-friendliness)	
BEN1	5.20 (1.35)
BEN2	5.21 (1.22)
BEN3	5.09 (1.44)
ZUG (accessibility)	
ZUG1	3.60 (1.49)
ZUG2	4.41 (1.48)
ZUG3	4.38 (1.46)
ITU (intention to use)	
ITU1	78.15 (20.53)
ITU2	70.34 (23.98)
ITU3	81.59 (17.95)

Pre-exposure assessments revealed high curiosity (NEU1: mean 6.04, SD 1.19) and low technology-related anxiety (ANG1: mean 2.38, SD 1.58). Following the storyboard intervention, participants rated the proposed system as generally useful (NÜT1: mean 5.99, SD 1.11) and user-friendly (BEN1: mean 5.20, SD 1.35), with only moderate skepticism. Accessibility received more varied evaluations (ZUG1: mean 3.60, SD 1.49).

We measured the ITU via a 0‐100 visual analog scale. Participants reported moderately high usage intentions, with item means ranging from 70.34 (SD 23.98) to 81.59 (SD 17.95) across the three ITU items.

#### Sociodemographic Variation in Intention to Use

**Figure 3.**
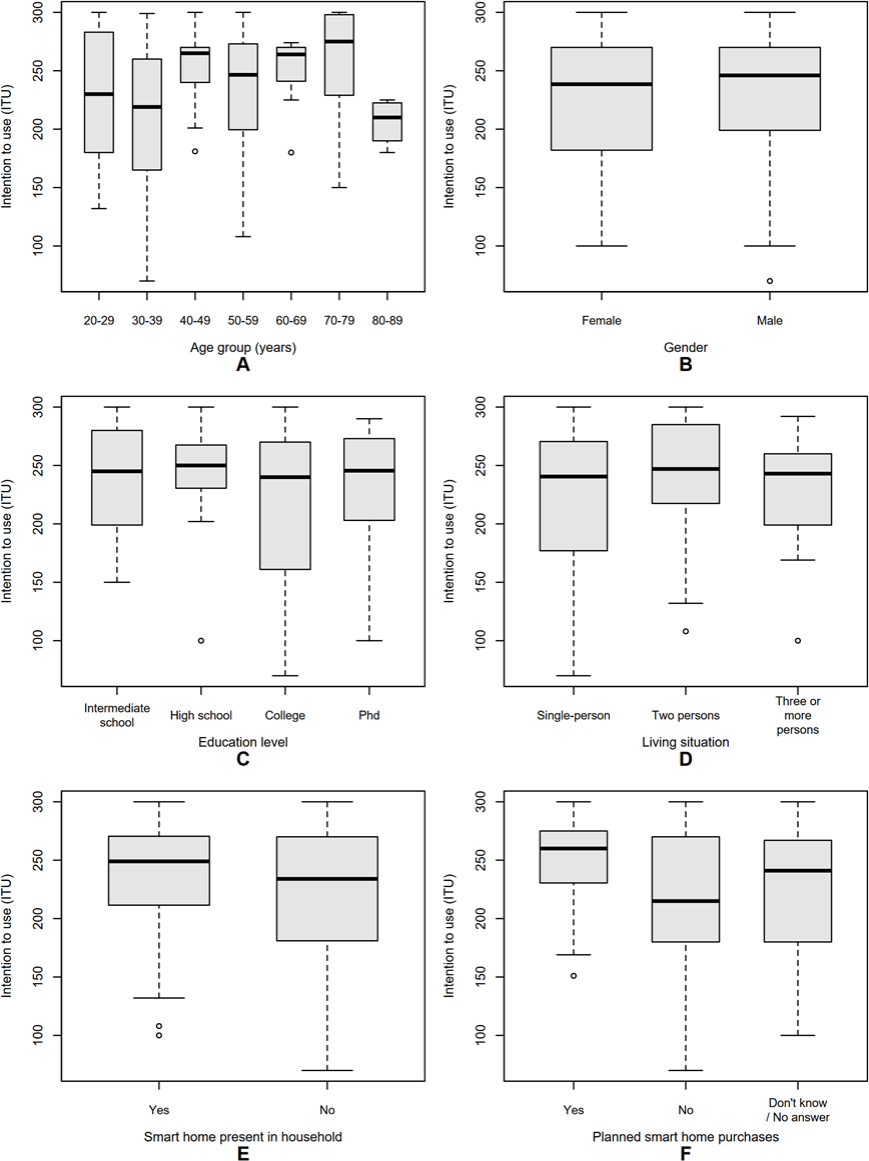
Distribution of intention to use (ITU) scores across sociodemographic and contextual subgroups. Panels show median, IQR, and outliers for (A) age group, (B) education level, (C) gender, (D) living situation, (E) planned smart-home purchases, and (F) smart-home presence in the household.

Before conducting the latent-variable analyses, we performed exploratory comparisons to examine whether ITU differed across sociodemographic and contextual subgroups. Figure 3 displays the distribution of ITU scores by age group, education level, gender, living situation, and smart-home–related characteristics (ie, presence of smart-home devices in the household and planned future purchases).

Across age categories (Figure 3A), median ITU scores appeared largely comparable, with slightly higher intentions in the youngest (20‐29 years) and older (70‐79 years) participants, but no consistent age-related trend. Regarding smart-home–related factors, participants who already had smart-home devices in their household (Figure 3E) tended to report slightly higher ITU scores than those without such devices, although the spread of values was wide and overlapping. Similarly, those who planned to purchase smart-home technologies in the near future (Figure 3F) reported somewhat elevated intentions relative to those without purchase plans, but again without clear statistical separation. Given the absence of significant mean-level differences in ITU across sociodemographic subgroups, these variables were not included as exogenous predictors in the SEM.

Overall, these analyses indicated no meaningful sociodemographic bias in ITU across the examined groups. This supports the focus on psychological and attitudinal constructs as the main determinants in the subsequent latent-variable modeling.

#### Preliminary Reliability and Correlational Analyses

Before estimating the latent variable model, we examined reliability and zero-order associations among the observed constructs. All multi-item scales demonstrated good internal consistency, with Cronbach α values ranging from 0.72 (BEN) to 0.93 (ADL) (see Table 5).

**Table 5.** Internal consistency and bivariate associations of study constructs with intention to use.

Construct	Cronbach α	Correlation with ITU^a^	β coefficient	*P* value
ADL (activities of daily living)	0.930	−0.064	−1.265	.56
SOC (social compatibility)	0.838	0.369	2.512	.001
NEU (curiosity)	0.813	0.366	4.158	.001
ANG (anxiety)	0.828	−0.112	−1.341	.31
INT (interest)	0.869	0.236	2.515	.03
NÜT (usefulness)	0.784	0.600	7.230	<.001
SKE (skepticism)	0.762	−0.539	−6.824	<.001
BEN (user-friendliness)	0.720	0.238	6.204	.03
ZUG (accessibility)	0.780	0.441	6.661	<.001

a ITU: intention to use.

Bivariate correlations showed that NÜT (*r*=0.60, *P*<.001), ZUG (*r*=0.44, *P*<.001), SOC (*r*=0.37, *P*=.001), NEU (*r*=0.37, *P*=.001), INT (*r*=0.24, *P*=.03), and BEN (*r*=0.24, *P*=.03) each correlated positively with ITU. In contrast, SKE correlated negatively (*r*=–0.54, *P*<.001), while ANG (*r*=–0.11, *P*=.31) and ADL (*r*=–0.06, *P*=.56) showed small, nonsignificant associations.

Simple linear regressions mirrored these bivariate patterns: positive effects on ITU emerged for NÜT, ZUG, SOC, NEU, INT, and BEN (all *P*≤.03), whereas SKE exerted a significant negative effect (β=–6.82, *P*<.001). Neither ANG nor ADL reached statistical significance (*P*>.30).

Together, these findings supported the proposed directionality of the theoretical model and justified proceeding to latent-variable analyses.

#### Confirmatory Factor and Structural Equation Modeling

We applied a comprehensive confirmatory factor and SEM approach using a DWLS estimator with robust corrections, appropriate for ordered-categorical indicators and the comparatively high number of observed variables per construct. The 9-factor measurement model, plus a 3-indicator factor for ITU, demonstrated an acceptable overall model fit on scaled indices (*χ*²_815_=1052.21, *P*<.001; CFI=0.928; TLI=0.920; RMSEA=0.059, 90% CI 0.048‐0.069; SRMR=0.107; Bentler-SRMR=0.0923) (Table 6).

**Table 6.** Scaled global model fit (DWLS^a^, robust) for the confirmatory factor and structural model (n=85).

Index	Value	Interpretation
Chi-square (*df*=815)	1052.21 (*P*<.001)	N/A^b^
CFI^c^	0.928	Acceptable
TLI^d^	0.920	Acceptable
RMSEA^e^	0.059 (90% CI 0.048‐0.069)	Good
SRMR^f^	0.107	Moderate
Bentler-SRMR	0.092	Acceptable given categorical indicators

a DWLS: diagonally weighted least squares.

b N/A: not applicable.

c CFI: comparative fit index.

d TLI: Tucker-Lewis index.

e RMSEA: root mean square error of approximation.

f SRMR: standardized root mean square residual.

Although both SRMR indices were slightly above conventional thresholds [43], simulation research indicates that SRMR tends to be inflated when using categorical estimators such as DWLS, particularly in complex models with many indicators [44]. The combination of CFI and TLI values close to 0.93 and an RMSEA below 0.06, given the categorical measurement level and the model’s complexity, supports an adequate and stable representation of the empirical covariance structure. All standardized factor loadings were positive, substantial, and statistically significant (*P*<.001), ranging from 0.46 to 0.99, with most exceeding 0.70. No cross-loadings or Heywood cases were detected, and the factor solution remained stable across estimation runs, confirming the stability of the measurement specification.

The SEM maintained an acceptable overall fit that closely mirrored the CFA solution and accounted for 66.1% of the variance in ITU (*R*²=0.661). Among the latent predictors, ZUG was the strongest positive determinant (β=0.325, *P*=.02). In contrast, SKE showed a negative association with ITU that approached but did not reach conventional significance levels (β=−0.336, *P*=.06). NÜT (β=0.357, *P*=.15) and SOC (β=0.129, *P*=.25) showed positive but nonsignificant trends, while BEN, INT, NEU, ANG, and ADL displayed no meaningful associations (|β|≤0.26, all *P*>.10; Table 7). A comparison between the full model (with all 9 predictors) and a reduced model (retaining only significant predictors) revealed minimal differences in fit indices (ΔCFI=−0.003; ΔRMSEA=0.004; ΔSRMR=0.004). Although a nested model test indicated a statistically significant decrement in fit (*P*=.001), the negligible changes in practical fit indices suggest that retaining theoretically motivated but statistically nonsignificant constructs did not materially distort the overall model.

**Table 7.** Standardized path coefficients predicting intention to use (scaled SEM^a^ and DWLS^b^).

Predictor → ITU (intention to use)	β (std)	SE	*z* score	*P* value	Significance
ZUG (accessibility)	0.325	0.239	2.33	.02	Significant
SKE (skepticism)	−0.336	0.312	−1.85	.06	Marginal
NÜT (usefulness)	0.357	0.431	1.42	.15	—^c^
SOC (social compatibility)	0.129	0.191	1.16	.25	—
BEN (user-friendliness)	−0.164	0.321	−0.87	.38	—
INT (interest)	0.070	0.323	0.37	.71	—
NEU (curiosity)	0.002	0.373	0.01	.99	—
ANG (anxiety)	−0.005	0.416	−0.02	.98	—
ADL (activities of daily living)	−0.255	0.324	−1.35	.18	—

a SEM: structural equation modeling.

b DWLS: diagonally weighted least squares.

c Not applicable.

**Figure 4.**
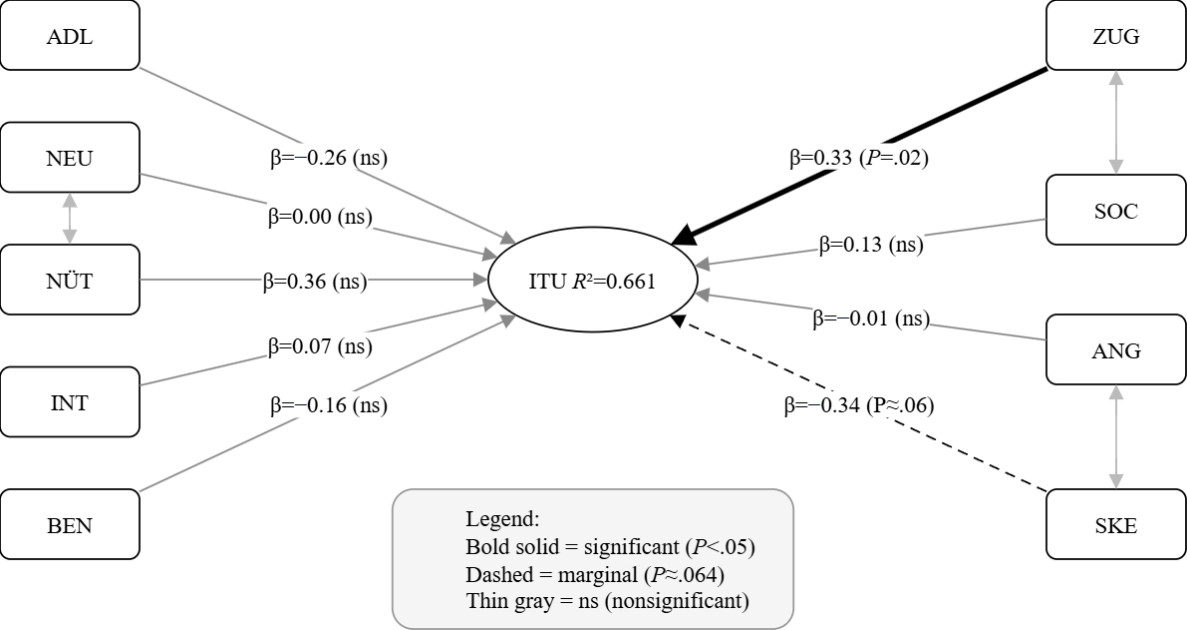
Structural equation model predicting intention to use (ITU) from psychological, functional, and social determinants. ADL: activities of daily living; ANG: anxiety; BEN: user-friendliness; INT: interest; ITU: intention to use; NEU: curiosity; NÜT: usefulness; SKE: skepticism; SOC: social compatibility; ZUG: accessibility.

The final structural equation model is depicted in Figure 4, illustrating the pattern of relationships among the 9 latent predictors and ITU. All exogenous latent constructs were allowed to covary, controlling for shared variance among psychological, functional, and social factors. These correlations are depicted as curved double-headed arrows in the structural model but were not interpreted further, as they were not part of the primary hypotheses.

To assess the robustness of the structural findings, we conducted several complementary diagnostics. To ensure that the constructs included in the latent-variable model were empirically distinct and not affected by multicollinearity, we performed additional post hoc diagnostics. The latent factor correlations extracted from the CFA model indicated satisfactory discriminant validity, as none of the interfactor correlations approached unity and all remained well below critical thresholds (|*r*|<0.85). This supports the conceptual distinctness of the modeled constructs.

Variance inflation factors (VIF) confirmed the absence of problematic multicollinearity, with values ranging from 1.24 to 1.81 (all VIF<5), indicating that the predictors were sufficiently independent. Alternative model specifications (eg, method factors, item parceling) provided no meaningful improvement and were not retained.

The findings provide a coherent picture of the psychological and contextual factors underlying intention to use. The next section discusses their theoretical and practical implications.

## Discussion

### Principal Findings

This mixed methods study identified key drivers and barriers of SHERS adoption by combining domain analysis, scenario-based design, and SEM.

The structural model explained two-thirds of the variance in behavioral intention (*R*²=0.66), underscoring the strong explanatory power of psychological and usability-related factors. Among all predictors, ZUG had the strongest positive effect on intention to use, emphasizing that participants’ perception of how easily the technology can be set up and integrated matters most for adoption. Conversely, SKE showed a negative association with intention that approached statistical significance (*P*=.06), suggesting that residual doubt about reliability, privacy, or false alarms may reduce willingness to adopt. Other theoretically relevant constructs such as NÜT and SOC displayed positive but nonsignificant trends, indicating that their effects may be more indirect or context dependent.

These findings suggest that beyond functional utility, the perceived ease of obtaining and setting up the technology may be more relevant to adoption intentions than its perceived benefits alone.

It is important to note that only ZUG reached conventional statistical significance as a predictor of intention to use. The negative trend for SKE (*P*=.06) is suggestive but does not meet the conventional threshold. All remaining constructs were nonsignificant in the multivariate model, despite several showing meaningful bivariate associations with intention to use. This pattern is consistent with the limited statistical power of the sample (n=85) to detect moderate effects among multiple correlated predictors and may also partly reflect the storyboard-based evaluation format, which limits experiential assessment of constructs such as BEN. Accordingly, interpretations involving nonsignificant predictors should be considered exploratory.

### Integration of Qualitative and Quantitative Findings

Methodological triangulation allowed cross-validation of qualitative and quantitative findings.

The quantitative analysis identified ZUG as the strongest predictor of intention to use. This finding directly mirrors qualitative themes: Expert 3 (unit leader) noted that “technical setup is complex and costly,” stressing that incentives or mandates would be necessary. Experts also stressed integration with existing infrastructure, proposing connections between “smart-watches, home emergency systems, and fire alarm systems” [Expert 3]. These convergent insights suggest accessibility represents a reliable determinant of adoption.

SKE was a negative predictor (*P*=.06), reflecting concerns about reliability, false alarms, and data privacy. Expert 1 (paramedic) proposed a “filter function to distinguish false alarms from actual emergencies” and mechanisms to “allow user intervention to cancel the alarm.” Expert 2 (dispatcher) emphasized that cameras should only be activated “after explicit request and consent.” These findings suggest skepticism is rooted in concrete operational and privacy concerns.

NÜT showed a positive but nonsignificant effect despite strong bivariate correlations. Experts recognized value for “vulnerable groups” [Expert 3], yet emphasized that determining whether “it is truly an emergency” should remain a “decision to be made by the incident commander” [Expert 3], and that “situational assessment by people on site is necessary” [Expert 2]. This tension may explain the attenuated effect. However, this interpretation remains exploratory, as the nonsignificant path coefficient may also reflect insufficient statistical power to detect a moderate effect at n=85, or measurement limitations arising from the storyboard-based evaluation format.

An overarching theme concerns the balance between automation and human control. Experts consistently emphasized that “human decision-making competence cannot be replaced” [Expert 1] and that “technology should initially only propose a classification” [Expert 1]. Expert 3 cautioned that “weighing up ‘deploy or not’ under the influence of data or a second opinion is highly critical.” These insights explain why both accessibility and low skepticism are central to acceptance.

Triangulation strengthens confidence in results while revealing contextual nuances.

### Comparison With Prior Work

The identified role of accessibility is consistent with findings from previous acceptance studies in digital health and smart-home contexts, where usability barriers frequently outweighed perceived benefits in determining behavioral intention [45,46].

The negative association between skepticism and intention mirrors earlier observations in related domains such as telemonitoring and artificial intelligence–assisted care [1,9]. Concerns about data privacy, false alarms, and perceived loss of control are well-documented inhibitors of trust in automated systems. The present results suggest that skepticism may reduce adoption intentions, although the association did not reach conventional significance (*P*=.06). This pattern is consistent with prior findings and points to trust calibration as a relevant design consideration.

Unlike some prior studies that found strong demographic effects, such as age-related declines in technology acceptance or gender-specific differences in curiosity and anxiety [47-49], the current analysis revealed no significant sociodemographic bias in intention to use. This suggests that, when users are adequately informed and exposed to a concrete system concept, psychological and design-related factors may play a larger role than demographic predispositions in shaping adoption intentions. This aligns with recent evidence indicating that participatory exposure and scenario-based demonstrations can reduce pre-existing reservations toward unfamiliar technologies [50].

### Practical Implications

From a design perspective, the findings highlight the importance of making accessibility tangible through intuitive setup, clear user guidance, and compatibility with existing smart home ecosystems. Developers should focus on reducing friction points such as complex configuration, opaque data handling, or unclear error feedback.

Addressing skepticism requires trust-building measures that communicate reliability and data protection standards. Clear alert protocols, privacy assurances, and demonstrated reliability under real conditions can increase confidence in automated emergency technologies. For policymakers and emergency service providers, these findings support the case for public communication and certification frameworks focused on safety and interoperability.

The consistency between expert and user perspectives observed in this study supports co-design approaches that bring emergency professionals and end users together early in the development process. Such collaboration can connect technical, psychological, and organizational perspectives and support the wider adoption of SHERS.

### Limitations

Several limitations should be considered. First, the quantitative evaluation relied on a conceptual rather than interactive prototype presented through annotated storyboards. Although this format is suitable for early-phase acceptance testing [34], it may have limited participants’ ability to evaluate usability and reliability. Constructs that depend on direct interaction experience, particularly BEN, may be underestimated or imprecisely captured when assessed on the basis of visual scenarios alone. This could partly explain the nonsignificant effect of BEN in the structural model, as participants lacked the hands-on experience needed to form differentiated usability judgments. Conversely, constructs reflecting general attitudes, such as curiosity or skepticism, are less likely to be affected by prototype fidelity. Future studies should use functional or simulated prototypes to validate these findings under more realistic conditions.

Second, although DWLS estimation is robust to smaller samples with ordinal data, the sample size (n=85) limits generalizability and the ability to test subgroup interactions. The recruitment strategy relied on housing cooperatives and senior advisory councils, which may have introduced selective bias by overrepresenting individuals with existing institutional ties and underrepresenting isolated, low-income, or digitally disengaged populations. These groups may stand to benefit most from SHERS but are typically harder to reach through organizational channels. The sample was also skewed toward higher educated (44% college degree) and male (65%) participants, which may affect the transferability of findings. Individuals with limited digital literacy or those living in underresourced housing conditions may hold different attitudes toward automated emergency systems. Larger and more diverse samples, including targeted recruitment of digitally excluded and lower-income populations, are needed to confirm the stability and generalizability of the observed effects.

The limited sample size also increases the risk of type II errors, particularly for predictors with moderate effect sizes. Several constructs with meaningful bivariate associations with intention to use, such as NÜT (*r*=0.60), did not reach significance in the multivariate model. While this pattern may partly reflect shared variance among correlated predictors, insufficient power to detect moderate effects in the presence of 9 simultaneous paths cannot be ruled out.

Third, self-reported data may have introduced social desirability bias. Behavioral or longitudinal observations could strengthen future analyses.

Finally, contextual variables (cost, connectivity, and interoperability) were not included and should be addressed in future research.

### Future Research

Future research should examine long-term acceptance trajectories and how trust develops during actual system use.

Cross-stakeholder studies should examine how acceptance differs between end users, emergency professionals, and service operators.

Evaluating artificial intelligence–driven emergency systems under field conditions will be necessary for safe deployment.

### Conclusions

This study examined the psychological and functional determinants of SHERS acceptance. Accessibility was the strongest statistically significant predictor of intention to use, while skepticism showed a negative trend that suggests it may function as a barrier to adoption. These results indicate that technological development alone is insufficient without user-centered design, open communication, and strategies that address adoption concerns early.

The mixed methods approach allowed us to connect design-level insights with statistical evidence on acceptance factors. Taken together, the findings offer practical orientation for developing emergency technologies that are trusted, usable, and compatible with the living environments of vulnerable populations.

## Supplementary material

10.2196/93003 Multimedia Appendix 1 Expert interview materials, including the semistructured interview guide and thematic mind maps from phase 1 domain analysis.

10.2196/93003 Multimedia Appendix 2 Qualitative reporting and design artifacts, including the COREQ checklist for phase 2 workshops and design requirements derived from scenario-based brainwriting sessions.

10.2196/93003 Multimedia Appendix 3 Survey instrument and CHERRIES checklist for phase 3 acceptance evaluation, including the adapted Technology Usage Inventory questionnaire.

## Abbreviations

ADL: activities of daily living
ANG: anxiety
BEN: user-friendliness
CFA: confirmatory factor analysis
CFI: comparative fit index
CHERRIES: Checklist for Reporting Results of Internet E-Surveys
COREQ: Consolidated Criteria for Reporting Qualitative Research
DWLS: diagonally weighted least squares
INT: interest
ITU: intention to use
JARS-MMARS: Mixed Methods Article Reporting Standards
NEU: curiosity (neugier)
NÜT: perceived usefulness
RMSEA: root mean square error of approximation
SEM: structural equation modeling
SHERS: smart home emergency response systems
SKE: skepticism
SOC: social compatibility
SRMR: standardized root mean square residual
TLI: Tucker-Lewis index
TUI: Technology Usage Inventory
VIF: variance inflation factor
ZUG: accessibility
