# Supplementary material for: User Acceptance of Smart Home Emergency Response Systems: Mixed Methods Study
Source: JMIR Hum Factors. 2026 Apr 20;13:e93003. doi: 10.2196/93003 (PMC13094802; doi:10.2196/93003)

**Multimedia Appendix 1.** Expert Interview Materials (Phase 1)

**A. Interview Guide**

**Overview**

Semi-structured interviews were conducted with three domain experts from the emergency response chain: a paramedic (emergency medical services), a fire department unit leader, and a control center dispatcher. The interviews followed a problem-centered approach, combining open-ended exploration with targeted probes on predefined topics.

**Interview Format**

Each interview lasted approximately 60 to 90 minutes and was conducted remotely using a virtual whiteboard (Miro). Participants were invited to co-create digital mind maps during the session, visually documenting their workflows, challenges, and ideas. This participatory method encouraged active engagement and facilitated the emergence of themes that might not surface in traditional verbal-only interviews.

**Opening Statement**

*The following statement was used to introduce the interview topic:*

*"This project aims to optimize the emergency response chain through intelligent, technology-based solutions. We are interested in your professional experiences and perspectives on how technology could support alerting, navigation, and coordination in emergency situations."*

**Thematic Areas and Guiding Questions**

The following thematic areas guided the conversation. Within each area, open-ended questions were posed, with follow-up probes used to explore emerging topics in greater depth.

1. **Qualified First Responders and Community Integration**

*Key question:* What are your experiences with community-based first responder programs?

- How are lay responders currently integrated into emergency response workflows?
- What information would first responders need before arriving at the scene?
- What challenges arise from involving non-professional helpers?

1. **Experiences With Fire Alarm Systems**

*Key question:* What are your experiences with automatic fire alarm systems in residential and commercial buildings?

- How reliable are current systems in distinguishing real emergencies from false alarms?
- What information is typically transmitted, and what additional data would be helpful?
- How do false alarms affect operational planning and resource allocation?

1. **Navigation Support During Approach**

*Key question:* What helps you navigate to the incident location efficiently?

- What challenges do you face when locating addresses or navigating to unfamiliar areas?
- How could real-time traffic or environmental data improve route planning?
- What role could smart infrastructure (e.g., traffic light preemption) play?

1. **Orientation Within and Around Buildings**

*Key question:* What helps you orient yourself once you arrive at a building?

- What information about building layout would be most useful?
- How could smart home or building systems support on-scene orientation?
- What are the main obstacles when entering residential buildings (e.g., locked doors, unclear layouts)?

1. **Automatic Alerting and System-Initiated Notifications**

*Key question:* How do you view the potential for automatic, sensor-triggered emergency alerts?

- What concerns would you have about systems that alert without human initiation?
- How should the balance between automation and human oversight be managed?
- What safeguards would be necessary to prevent misuse or system errors?

1. **Scope and Presentation of Information**

*Key question:* What type and amount of information would be most useful during dispatch and response?

- How should information be prioritized or filtered based on responder role?
- What are the risks of information overload versus information scarcity?
- How could patient health data (e.g., medication plans) be securely integrated?

1. **Building Access and Door-Opening Procedures**

*Key question:* What challenges do you face with building access during emergencies?

- How could smart lock systems facilitate authorized emergency entry?
- What security and liability concerns would need to be addressed?
- How is door access currently coordinated between fire services and police?

1. **Open Ideas and Future Possibilities**

*Key question:* Beyond the topics discussed, what other technological solutions could improve emergency response?

- Are there any innovations from other domains that could be adapted?
- What would your ideal future emergency response system look like?
- What barriers (technical, organizational, legal) need to be overcome?

**Closing**

At the end of each interview, participants were asked to review the co-created mind map, add any missing points, and highlight the three ideas or challenges they considered most important. The resulting mind maps served as both data artifacts and member-checked summaries of the interview content.

**Data Handling**

Mind maps were exported and analyzed thematically. Due to the participatory, visual nature of data collection, traditional verbatim transcription was not applicable. Instead, the structured mind map outputs served as the primary data source for thematic clustering and informed the development of personas and scenarios in Phase 2.

**B. Mind Map Outputs**

**Figure S1.** Mind Map: Emergency Medical Services Perspective (Paramedic).


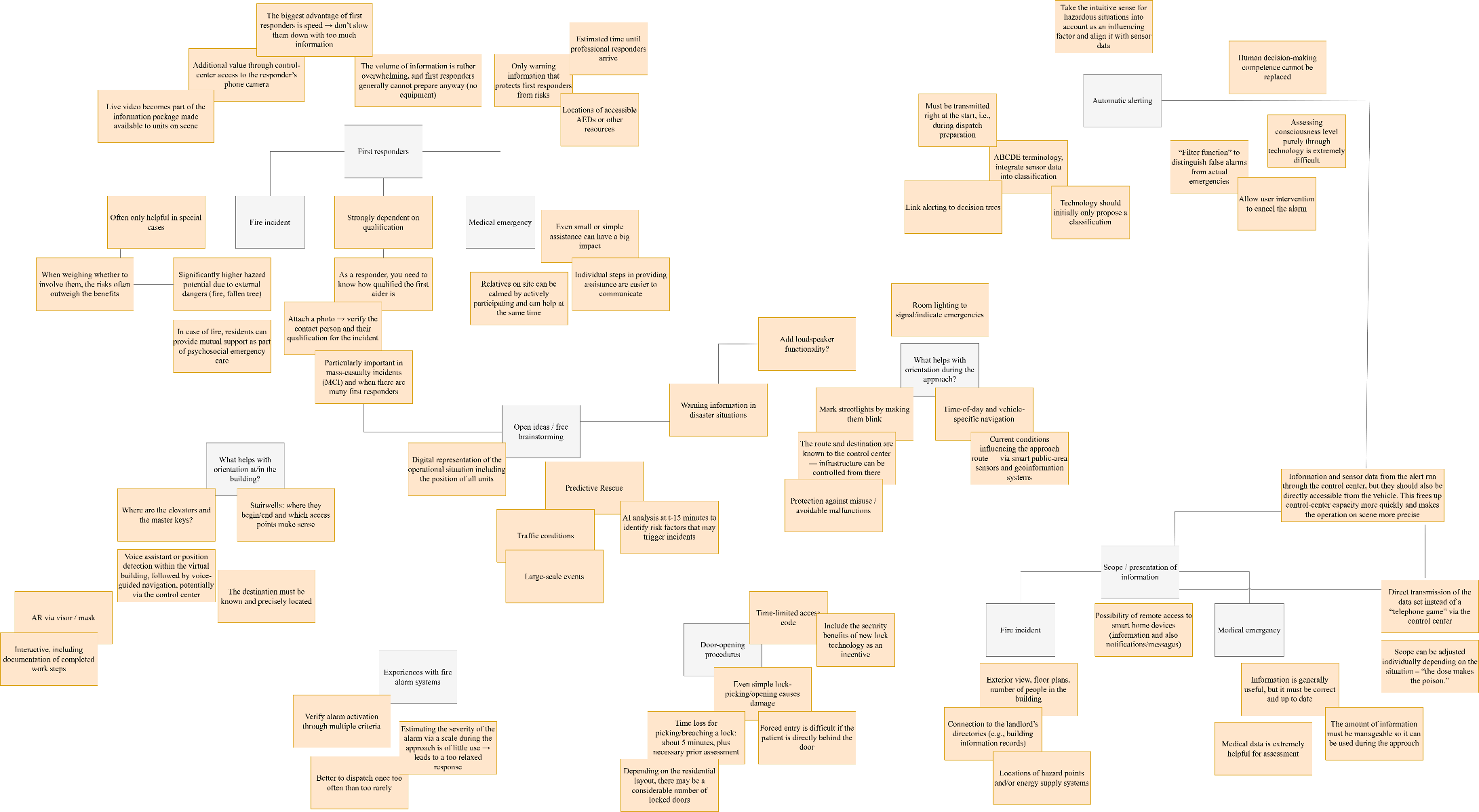


**Figure S2.** Mind Map: Control Center Perspective (Dispatcher).


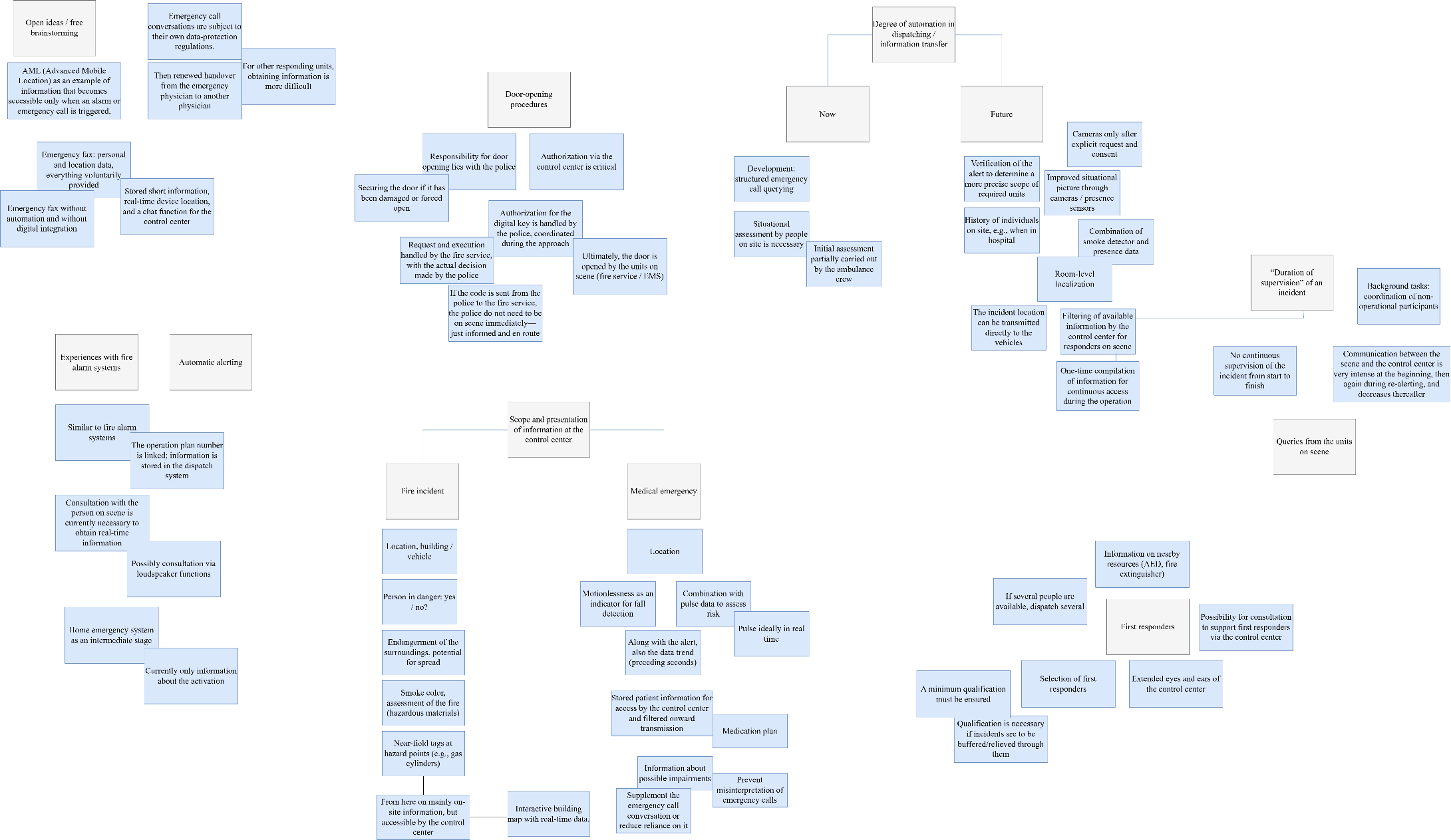


**Figure S3.** Mind Map: Fire Department Perspective (Unit Leader).


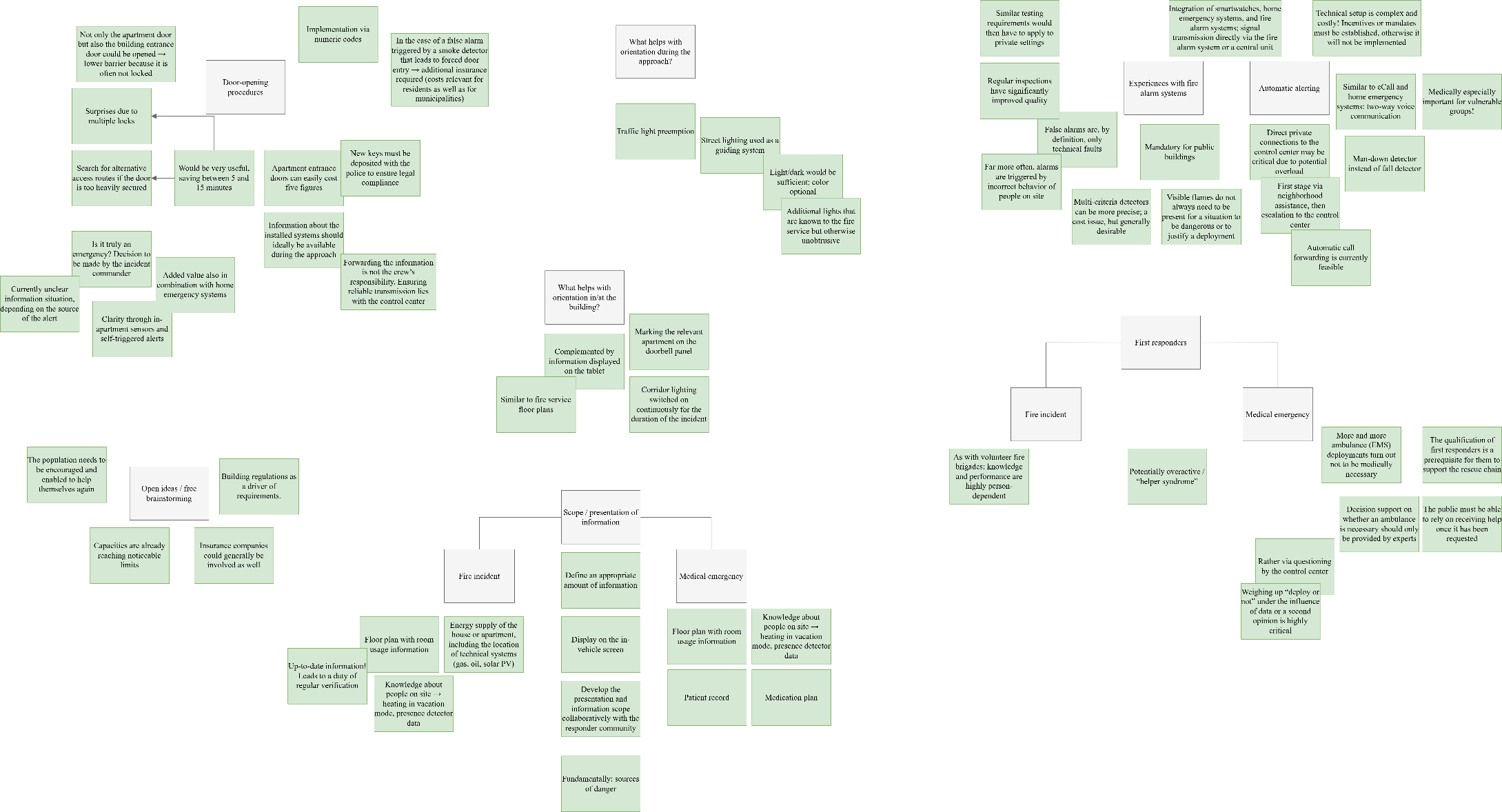

Supplement: Multimedia Appendix 1 [file humanfactors-v13-e93003-s001.docx]
