# Supplementary material for: User Acceptance of Smart Home Emergency Response Systems: Mixed Methods Study
Source: JMIR Hum Factors. 2026 Apr 20;13:e93003. doi: 10.2196/93003 (PMC13094802; doi:10.2196/93003)
Supplement: Multimedia Appendix 2 [file humanfactors-v13-e93003-s002.docx]

**Multimedia Appendix 2.** Qualitative Reporting and Design Artefacts.

**Table S1.** COREQ Checklist applied to Phase Two: Scenario-Based Design Workshops.

| **Domain / Item** | **Description / Response** | **Location in Manuscript** |
| --- | --- | --- |
| Domain 1: Research team and reflexivity |  |  |
| 1. Interviewer/facilitator | Workshops moderated by MP and AR. | Methods → Phase Two: Brainwriting With Domain Experts |
| 2. Credentials | MP and AR are researchers with experience in participatory and user-centered design. | — |
| 3. Occupation | Both moderators were research scientists at Fraunhofer ISST at the time of study. | — |
| 4. Gender | One male (MP), one female (AR). | — |
| 5. Experience and training | Both had prior experience conducting qualitative and participatory workshops in technology design contexts. | — |
| 6. Relationship established | No prior personal or professional relationship with participants. | — |
| 7. Participant knowledge of the interviewer | Participants were informed about the study’s aim and the facilitators’ institutional role before the session. | Methods → Phase Two: Brainwriting With Domain Experts |
| 8. Interviewer characteristics | Facilitators maintained a neutral, structured moderation role; potential bias minimized by independent analysis by both researchers. | Methods → Data Processing and Analysis |
| Domain 2: Study design |  |  |
| 9. Methodological orientation | Qualitative content analysis applied to structured brainwriting outputs (Scenario-Based Design framework). | Methods → Data Processing and Analysis |
| 10. Sampling | Purposive sampling based on relevant professional roles within the emergency response domain. | Methods → Phase Two: Brainwriting With Domain Experts |
| 11. Method of approach | Participants recruited via professional networks through the Dortmund Fire Department. | Methods → Phase Two: Brainwriting With Domain Experts |
| 12. Sample size | Two workshops, each with six participants. | Methods → Phase Two: Brainwriting With Domain Experts |
| 13. Non-participation | All invited participants attended; no withdrawals reported. | — |
| 14. Setting of data collection | Conducted online via Conceptboard, allowing real-time collaboration. | Methods → Data Processing and Analysis |
| 15. Presence of non-participants | Only invited participants and facilitators were present. | — |
| 16. Description of sample | Emergency professionals (first responders, dispatchers, academic experts in aging, HMI, socio-technical systems). | Methods → Phase Two: Brainwriting With Domain Experts |
| 17. Interview guide | Structured brainwriting template with open prompts on system needs, usability, and integration. | Methods → Phase Two: Brainwriting With Domain Experts |
| 18. Repeat sessions | No repeat sessions; feedback incorporated iteratively between the two workshops. | Methods → Phase Two: Scenario Iteration and Consolidation |
| 19. Recording | No audio or video recording; only digital whiteboard artefacts were stored. | Methods → Data Processing and Analysis |
| 20. Field notes | All written contributions preserved within Conceptboard as digital sticky notes. | Methods → Data Processing and Analysis |
| 21. Duration | Approximately 50–60 minutes per workshop (10 min introduction, 10 min method briefing, 30–40 min brainwriting). | Methods → Phase Two: Brainwriting With Domain Experts |
| 22. Data saturation | Not applicable; workshops aimed at breadth of design inputs rather than theoretical saturation. | — |
| 23. Transcripts returned | Not applicable; written workshop outputs already participant-generated. | — |
| Domain 3: Analysis and findings |  |  |
| 24. Number of data coders | Two (MP and AR). | Methods → Data Processing and Analysis |
| 25. Description of coding tree | Codes derived inductively and clustered into higher-order categories (user needs, system functions, integration). | Methods → Data Processing and Analysis |
| 26. Derivation of themes | Themes emerged inductively from the data. | Methods → Data Processing and Analysis |
| 27. Software | Microsoft Excel used for coding and theme clustering. | Methods → Data Processing and Analysis |
| 28. Participant checking | Not performed due to exploratory, design-oriented nature of the workshops. | — |
| 29. Quotations presented | Representative idea clusters summarized narratively in the Results (Phase Two). | Results → Phase Two: Scenario Development & Requirements Engineering |
| 30. Data and findings consistent | Findings directly traceable to coded idea clusters documented on Conceptboard. | Results → Phase Two |
| 31. Clarity of major themes | Major themes presented as system requirements and design implications. | Results → Phase Two: Findings From Scenario-Based Brainwriting |
| 32. Clarity of minor themes | Minor or divergent ideas incorporated into subsequent scenario iterations. | Results → Phase Two: Scenario Iteration and Consolidation |

**TableS2.** Design Requirements Derived From Scenario-Based Design Workshops.

*Design requirements were synthesized from two brainwriting workshops (6-3-5 method). Workshop 1 (n=6 first responders) focused on technical system capabilities. Workshop 2 (n=6 socio-technical experts) addressed usability and acceptance. Requirements are categorized into functional requirements (FR) and non-functional requirements (NFR).*

**Functional Requirements**

| ID | Category | Requirement Description | Source |
| --- | --- | --- | --- |
| FR1 | Detection | The system shall detect emergencies through body-worn sensors (smartwatch, insulin pump, CGM) and transmit alerts automatically. | WS1 |
| FR2 | Detection | The system shall detect emergencies through ambient home sensors (smoke detectors, motion sensors) and initiate alerts based on predefined rules. | WS1 |
| FR3 | Verification | The system shall allow users to cancel false alarms before transmission through voice assistant, dead man's switch, or manual override. | WS1, WS2 |
| FR4 | Information | The system shall automatically attach user information to emergency calls (location, medical history, medication, occupants). | WS1 |
| FR5 | Information | The system shall allow users to configure personal emergency information during setup (conditions, contacts, access codes). | WS1, WS2 |
| FR6 | Notification | The system shall notify neighbors and community first responders (Mobile Retter) simultaneously with professional services. | WS1 |
| FR7 | Notification | The system shall alert first responders based on qualification, proximity, and arrival time. | WS1 |
| FR8 | Remote Access | The system shall enable dispatch centers to remotely activate smart home devices for situational awareness. | WS1 |
| FR9 | Control | The system shall control connected devices in emergencies (unlock doors, disable stove, activate lighting for wayfinding). | WS1 |
| FR10 | Navigation | The system shall provide optimized routing incorporating real-time traffic, construction, and weather. | WS1 |
| FR11 | Navigation | The system shall enable visual and acoustic wayfinding aids (blinking lights, markers, smart streetlights). | WS1 |
| FR12 | Communication | The system shall establish communication channels between first responders, dispatch, and on-scene helpers. | WS1 |
| FR13 | Aftercare | The system shall provide first responders access to psychological support following critical incidents. | WS1 |

**Non-Functional Requirements**

| ID | Category | Requirement Description | Source |
| --- | --- | --- | --- |
| NFR1 | Usability | Interface shall be self-explanatory; critical functions directly accessible without complex menus. | WS2 |
| NFR2 | Usability | The system shall use familiar interaction paradigms based on users' existing technology experience. | WS2 |
| NFR3 | Usability | The system shall provide multiple input modalities (touch, voice, buttons) for varying capabilities. | WS2 |
| NFR4 | Accessibility | Interface shall adapt to users' changing cognitive and physical abilities over time. | WS2 |
| NFR5 | Learnability | The system shall include tutorials, video guides, and peer-based learning support. | WS2 |
| NFR6 | Learnability | Initial setup shall be conducted with personal assistance from trained personnel or family. | WS2 |
| NFR7 | Acceptance | The system shall provide everyday utility beyond emergencies to increase adoption motivation. | WS2 |
| NFR8 | Acceptance | Design shall avoid stigmatizing users; framing should emphasize empowerment and independence. | WS2 |
| NFR9 | Acceptance | Benefits shall be communicated through concrete use cases rather than fear-based messaging. | WS2 |
| NFR10 | Privacy | The system shall transparently display data collection and allow users to control sharing preferences. | WS2 |
| NFR11 | Privacy | Health data shall be accessible only to emergency personnel, not insurance providers. | WS1, WS2 |
| NFR12 | Configurability | The system shall support modular configuration based on individual needs. | WS2 |
| NFR13 | Configurability | Settings shall allow reassessment following changes in health or living situation. | WS2 |
| NFR14 | Reliability | The system shall complement, not replace, existing emergency infrastructure (112). | WS2 |
| NFR15 | Social | The system should facilitate neighborhood networks and mutual support beyond emergencies. | WS2 |
| NFR16 | Equity | Access shall not depend on financial resources or technical sophistication. | WS2 |

**Key Themes Across Both Workshops**

- Human Oversight: Final alerting decisions should involve human verification rather than fully autonomous action.
- False Alarm Prevention: Multi-sensor validation and user confirmation mechanisms are essential for acceptance.
- Transparent Data Handling: Clear communication about data usage builds trust; strict separation from commercial interests is mandatory.
- Everyday Value: Integration into daily routines increases acceptance; emergency-only systems face adoption barriers.
- Inclusive Design: Older adults are heterogeneous; solutions must accommodate diverse abilities and living situations.
- Social Embedding: Technology should strengthen rather than replace social networks and neighborhood connections.

**Figure S1.** Storyboard – Fire Scenario.

**
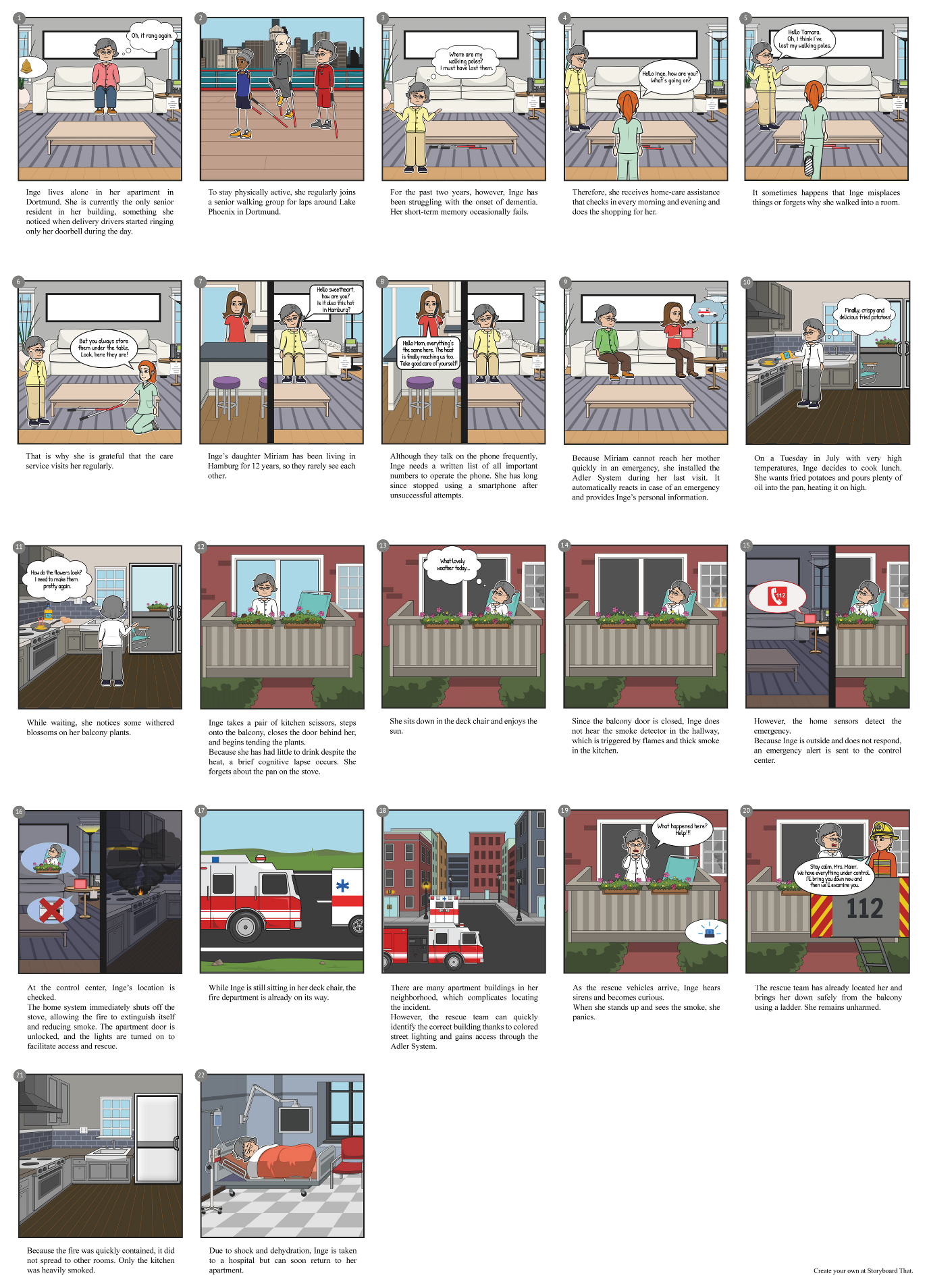
**

**Figure S2.** Storyboard – Medical Emergency Scenario.

**
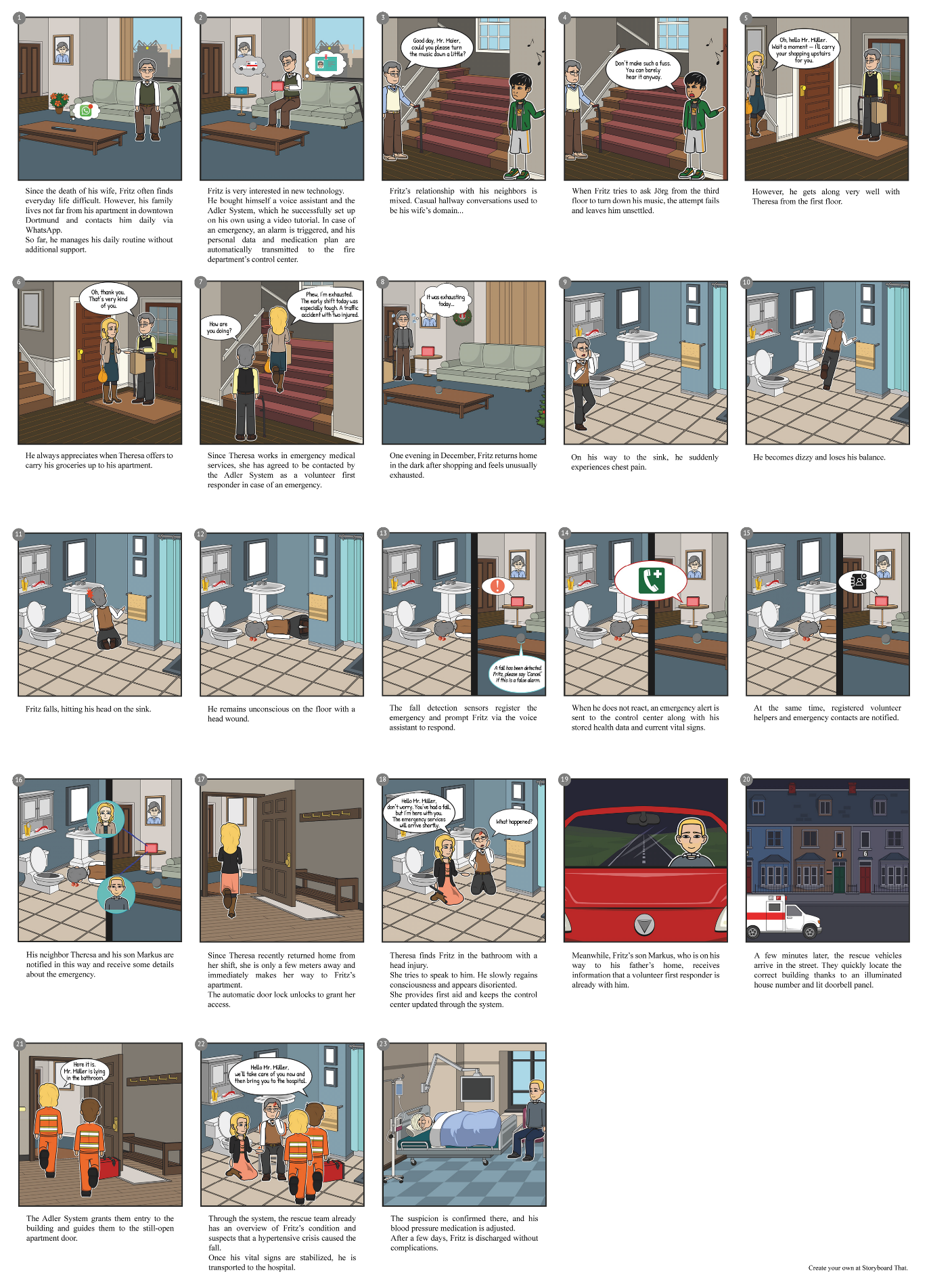
**
