# Supplementary material for: User Acceptance of Smart Home Emergency Response Systems: Mixed Methods Study
Source: JMIR Hum Factors. 2026 Apr 20;13:e93003. doi: 10.2196/93003 (PMC13094802; doi:10.2196/93003)
Supplement: Multimedia Appendix 3 [file humanfactors-v13-e93003-s003.docx]

**Multimedia Appendix 3.** Survey Instrument and CHERRIES Checklist (Phase 3).

**Table S1.** Questionnaire used in the online survey (Phase 3).

| **Section / Topic** | **Question (English)** | **Question (German)** | **Response Options / Scale** |
| --- | --- | --- | --- |
| **A. Demographic data** | To which of the following age categories do you belong? | Zu welcher der nachfolgenden Alterskategorien gehören Sie? | a. 19 or younger b. 20–29 c. 30–39 d. 40–49 e. 50–59 f. 60–69 g. 70–79 h. 80–89 i. 90 or older |
|  | Please indicate your gender. | Bitte geben Sie Ihr Geschlecht an. | a. Female b. Male c. Diverse |
|  | What is your highest school or university degree? | Was ist Ihr höchster Schul- oder Hochschulabschluss? | a. No degree b. Lower secondary school certificate c. Intermediate secondary school certificate d. University entrance qualification e. Bachelor’s degree f. Master’s degree / Diploma g. Doctorate / PhD |
|  | What is your current living situation? | Wie ist Ihre aktuelle Wohnsituation? | a. Single-person household b. Two persons c. Three or more persons d. Retirement / nursing home |
|  | Do you belong to any of the following aid organisations? *(multiple answers possible)* | Gehören Sie einer der folgenden Hilfsorganisationen an? *(Mehrfachauswahl möglich)* | a. Volunteer fire brigade b. Professional fire service c. Full-time emergency medical service d. Volunteer emergency medical service e. Technical relief agency (THW) f. Disaster relief service g. Police |
| **B. Experience with smart home applications** | Do you use smart home applications in your household? | Nutzen Sie Smart-Home-Anwendungen in Ihrem Haushalt? | a. Yes b. No |
|  | If yes, which smart home devices do you use in your household? *(multiple answers possible)* | Wenn ja, welche Smart-Home-Geräte nutzen Sie in Ihrem Haushalt? *(Mehrfachauswahl möglich)* | a. Lighting b. Heating c. Sockets d. Energy meters e. Alarm system f. Video surveillance g. Smoke detectors h. Locking system i. Home emergency call system j. Blinds / awnings k. Vacuum robot l. Garden devices m. Other (open input) |
|  | If yes, why do you use smart home applications? *(multiple answers possible)* | Wenn ja, warum nutzen Sie Smart-Home-Anwendungen? *(Mehrfachauswahl möglich)* | a. More comfort and quality of life b. Greater safety c. Energy efficiency d. Saving money e. Remote access f. Installation by landlord g. Saving time h. Enjoyment of technology i. Living independently for longer j. Other (open input) |
|  | Are you planning to purchase any smart home applications in the next 12 months? | Planen Sie, in den kommenden 12 Monaten Anschaffungen von Smart-Home-Anwendungen? | a. Yes b. No c. Not sure / no answer |
|  | If yes, which smart home devices do you plan to buy? *(multiple answers possible)* | Wenn ja, welche Smart-Home-Geräte planen Sie zu kaufen? *(Mehrfachauswahl möglich)* | a. Lighting b. Heating c. Sockets d. Energy meters e. Alarm system f. Video surveillance g. Smoke detectors h. Locking system i. Home emergency call system j. Blinds / awnings k. Vacuum robot l. Garden devices m. Other (open input) |
| **C. Functional and Psychological Factors** | | | |
| **Activities of Daily Living (ADL)** | While sitting on a chair, can you stand up without using your arms? | Können Sie auf einem Stuhl sitzend ohne Hilfe der Arme aufstehen? | 5-point Likert scale: 1 = I cannot perform this activity, 2 = I have major difficulties, 3 = I have moderate difficulties, 4 = I have slight difficulties, 5 = I have no difficulties |
|  | Can you walk briskly for several blocks? | Können Sie um mehrere Blocks flott gehen? | Same 5-point scale as above |
|  | Can you put on and take off a tight pullover and socks by yourself? | Können Sie einen engen Pulli und Socken allein aus- und anziehen? | Same 5-point scale as above |
|  | Can you walk down a flight of stairs without holding on? | Können Sie eine Treppe hinab gehen, ohne sich festzuhalten? | Same 5-point scale as above |
| **Social Compatibility (SOC)** | When I see others in difficulty, I spontaneously offer my help. | Wenn ich sehe, dass andere in Schwierigkeiten sind, biete ich spontan meine Hilfe an. | 6-point Likert scale: 1 = Does not apply at all, 6 = Fully applies |
|  | When you take care of others, this is usually seen as interference with their privacy. It is best to stay out of it. (reverse coded) | Wenn man sich um andere kümmert, wird dies doch meist als Einmischung in die Privatsphäre verstanden. Am besten, man hält sich da heraus. (invertiert) | Same 6-point scale as above |
|  | I believe that even one person alone can do a lot to help others. | Ich glaube, dass man auch als einzelner eine Menge bewegen kann, um anderen Menschen zu helfen. | Same 6-point scale as above |
|  | It is not really worth burdening yourself with other people’s problems. You can’t do anything about it anyway. (reverse coded) | Es lohnt im Grunde nicht, sich mit Problemen anderer zu belasten. Man kann sowieso nichts dagegen tun. (invertiert) | Same 6-point scale as above |
|  | As an individual, one cannot change social grievances anyway. (reverse coded) | Als einzelner kann man an sozialen Missständen doch nichts ändern. (invertiert) | Same 6-point scale as above |
|  | I regularly take care of “outsiders” in society (e.g., foreigners, refugees, people with disabilities, elderly, or sick people). | Ich kümmere mich regelmäßig um „Außenseiter“ der Gesellschaft (z. B. Ausländer, Asylanten, Behinderte, Alte, Kranke). | Same 6-point scale as above |
|  | I simply don’t have time to take care of others. (reverse coded) | Ich habe einfach keine Zeit, mich um andere zu kümmern. (invertiert) | Same 6-point scale as above |
|  | In my free time, I want to relax and have fun rather than deal with other people’s problems. (reverse coded) | In meiner Freizeit möchte ich mich erholen und Spaß haben und mich nicht auch noch mit Problemen anderer belasten. (invertiert) | Same 6-point scale as above |
|  | I can’t deal with other people’s problems; I already have enough of my own. (reverse coded) | Ich kann mich nicht mit den Problemen anderer belasten; ich habe genug mit mir selbst zu tun. (invertiert) | Same 6-point scale as above |
|  | What matters most to me at work is being able to help other people. | Bei meiner Arbeit ist mir vor allem wichtig, dass ich anderen Menschen helfen kann. | Same 6-point scale as above |
| **Technology Usage Inventory – Pre (NEU, ANG)** | I am curious about using this technology. | Ich bin neugierig auf die Verwendung dieser Technologie. | 7-point Likert scale: 1 = Does not apply, 7 = Applies completely |
|  | I often worry that new technical devices could overwhelm me. | Ich mache mir oft Sorgen darüber, dass mich neue technische Geräte überfordern könnten. | Same 7-point scale as above |
|  | I wanted to engage with this technology earlier. | Ich wollte mich schon früher mit dieser Technologie beschäftigen. | Same 7-point scale as above |
|  | When I am supposed to use a new technical device, I am initially distrustful. | Wenn ich ein neues technisches Gerät verwenden soll, bin ich erst mal misstrauisch. | Same 7-point scale as above |
|  | I strive to learn more about this technology. | Ich bin bestrebt, mehr über diese Technologie zu erfahren. | Same 7-point scale as above |
|  | It is hard for me to trust technical devices. | Mir fällt es schwer, technischen Geräten zu vertrauen. | Same 7-point scale as above |
|  | I have always been interested in using this technology. | Mich hat die Verwendung dieser Technologie schon immer interessiert. | Same 7-point scale as above |
|  | The idea of doing something wrong when using technical devices makes me anxious. | Die Vorstellung, bei der Verwendung technischer Geräte etwas falsch zu machen, macht mir Angst. | Same 7-point scale as above |
| **D. Post-Storyboard Evaluation and Intention to Use** | | | |
| **Technology Usage Inventory – Post (INT, NÜT, SKE, BEN, ZUG)** | I have acquired a lot of technical knowledge throughout my life. | Im Laufe meines Lebens habe ich mir viel technisches Wissen angeeignet. | 7-point Likert scale: 1 = Does not apply, 7 = Applies completely |
|  | Using this technology would make many things more comfortable. | Die Anwendung dieser Technologie würde vieles komfortabler machen. | Same 7-point scale as above |
|  | I think using this technology always involves a certain risk. | Ich denke, dass die Nutzung dieser Technologie immer mit einem gewissen Risiko verbunden ist. | Same 7-point scale as above |
|  | Using this technology is easy to understand. | Die Anwendung dieser Technologie ist leicht verständlich. | Same 7-point scale as above |
|  | When a new technical device comes on the market, I inform myself about it. | Wenn ein neues technisches Gerät auf den Markt kommt, informiere ich mich darüber. | Same 7-point scale as above |
|  | This technology would help me perform my daily tasks more conveniently. | Diese Technologie würde mir helfen, meine täglichen Aufgaben bequemer zu erledigen. | Same 7-point scale as above |
|  | I think this technology poses dangers to me. | Ich denke, dass diese Technologie Gefahren für mich birgt. | Same 7-point scale as above |
|  | Overall, using this technology is easy. | Die Anwendung dieser Technologie ist insgesamt einfach. | Same 7-point scale as above |
|  | I think almost everyone can afford this technology. | Ich denke, dass sich diese Technologie fast jeder leisten kann. | Same 7-point scale as above |
|  | I always try to get up-to-date information on new technological developments. | Ich versuche immer, aktuelle Informationen über neue technische Entwicklungen zu bekommen. | Same 7-point scale as above |
|  | If I could afford this technology, I would buy it. | Könnte ich mir diese Technologie leisten, würde ich sie mir anschaffen. | Same 7-point scale as above |
|  | This technology would disrupt my daily routine. | Diese Technologie würde meine Alltagsroutine stören. | Same 7-point scale as above |
|  | Using this technology is complicated. | Die Anwendung dieser Technologie ist kompliziert. | Same 7-point scale as above |
|  | I think this technology is generally accessible to everyone. | Ich denke, dass diese Technologie grundsätzlich für jeden zugänglich ist. | Same 7-point scale as above |
|  | I inform myself about technological developments. | Ich informiere mich über technologische Entwicklungen. | Same 7-point scale as above |
|  | This technology would give me more confidence in managing my daily tasks. | Diese Technologie würde mir mehr Sicherheit dabei geben, meine alltäglichen Aufgaben zu erfüllen. | Same 7-point scale as above |
|  | Using this technology would bring me more disadvantages than advantages. | Die Anwendung dieser Technologie würde mir mehr Nachteile als Vorteile bringen. | Same 7-point scale as above |
|  | I think setting up this technology requires little effort. | Ich denke, dass die Einrichtung dieser Technologie mit wenig Aufwand verbunden ist. | Same 7-point scale as above |
| **Intention to Use (ITU)** | Would you use this technology? | Würden Sie diese Technologie nutzen? | Visual analog scale (0–100): “Would definitely not use” → “Would definitely use” |
|  | Would you purchase this technology? | Würden Sie sich diese Technologie anschaffen? | Same visual analog scale as above |
|  | Would you like to have access to this technology? | Würden Sie Zugang zu dieser Technologie haben wollen? | Same visual analog scale as above |

*Source: Adapted from Technology Usage Inventory (Kothgassner et al., 2013)*

**Table S2.** CHERRIES checklist for reporting web-based surveys.

| **Item Category** | **Checklist Item** | **Explanation** |
| --- | --- | --- |
| Design | Describe survey design | The study was conducted among a diverse cross section of the German population using an online survey. The sample is not a convenience sample. The survey followed a cross-sectional, non-experimental design. |
| IRB (Institutional Review Board) approval and informed consent process | IRB approval | The ethics committee of the university of Witten/Herdecke approved the survey on 07.09.2022 under the protocol code: S-152/2022. |
|  | Informed consent | Participants were informed on the survey welcome page. Information and consent to participate in Research Study have been provided in German. The study participants remain anonymous. By clicking the checkbox, consent was confirmed. Participation was voluntary, and respondents could withdraw at any time without providing reasons. |
|  | Data protection | The survey was hosted and all data were stored on its own secure server. No personal information was linked to the survey results in any way. Data were stored on university servers compliant with GDPR (EU 2016/679). |
| Development and pre-testing | Development and testing | The methods used (TUI, ADL, SOC) consists of mainly standardised questions, which have been proved in various previous studies. A pilot survey was administered to a small sample of participants (n=5). The pretest assessed clarity and technical functionality. Feedback was used to make minor adjustments to the survey instrument. |
| Recruitment process and description of the sample having access to the questionnaire | Open survey versus closed survey | The survey was an open survey. |
|  | Contact mode | Participants were primarily contacted through associated housing corporations and senior advisory councils. Furthermore, the study was shared by the Dortmund Fire Department on X, Facebook and LinkedIn. Participants were able to share the link to the study. |
|  | Advertising the survey | The study was announced by the contact persons in the associated housing corporations, senior advisory councils, and the Dortmund fire department. |
| Survey administration | Web/E-mail | The survey was hosted on its own web server by the University Witten/Herdecke in Germany, using the software LimeSurvey. |
|  | Context | The survey landing page was publicly accessible and distributed through an URL. This ensured that participants were able to share the survey. No password was required for access. |
|  | Mandatory/voluntary | The survey was completely voluntary. Users could access the landing page without completing the survey. |
|  | Incentives | No incentives were offered to participants. |
|  | Time/Date | The survey period was from 01.10.22 to 30.06.2023. |
|  | Randomisation of items or questionnaires | The survey items were not randomised. |
|  | Adaptive questioning | Three adaptive questions were included in Section B of the survey, following the use and adoption of smart homes. For example, follow-up items on planned purchases were only displayed to respondents indicating prior smart home use. |
|  | Number of Items | The survey included 53 items across four sections.  Section A: 5 questions  Section B: 5 questions  Section C: 22 standardised questions  Section D: 21 standardized questions |
|  | Number of screens (pages) | One welcome page and 8 pages with survey items. |
|  | Completeness check | Most of the survey items were mandatory, and respondents were asked to complete outstanding items before leaving the survey page. |
|  | Review step | Participants were able to review and change their answers by clicking the Back button. |
| Response rates | Unique site visitor | Cookies were used to prevent multiple entries; therefore, unique site visitors could be tracked indirectly via cookies but not stored permanently. |
|  | View rate (ratio of unique survey visitors/unique site visitors) | Not measured. |
|  | Participation rate (ratio of unique visitors who agreed to participate/unique first survey page visitors) | Not measured. |
|  | Completion rate (ratio of users who finished the survey/users who agreed to participate) | Section A: 128/128 = 100%  Section B: 111/128 = ~ 87%  Section C: 98/128 = ~ 76%  Section D: 85/128 = ~ 66% |
| Preventing multiple entries from the same individual | Cookies used | A cookie has been set to prevent repeated participation from the same device. |
|  | IP check | No IP controls were used. |
|  | Log file analysis | Log file analysis was used for time stamps and completion patterns. |
|  | Registration | Not necessary since the survey was an open survey. |
| Analysis | Handling of incomplete questionnaires | Only completed questionnaires were included in the analysis. |
|  | Questionnaires submitted with an atypical timestamp | Not used. |
|  | Statistical correction | No statistical correction procedures or weightings were used in the analysis. |

*Source: Adapted from CHERRIES checklist (Eysenbach, 2004).*
